# Wearable Self‐Powered Pressure Sensors Based on alk‐Ti_3_C_2_T_x_ Regulating Contact Barrier Difference for Noncontact Motion Object Recognition

**DOI:** 10.1002/advs.202416504

**Published:** 2025-02-07

**Authors:** Yanan Xiao, Qi Pu, Chenxing Wang, Xiaoteng Jia, Shixiang Sun, Quan Jin, Xiaolong Wang, Bin Wang, Peng Sun, Fangmeng Liu, Geyu Lu

**Affiliations:** ^1^ State Key Laboratory of Integrated Optoelectronics College of Electronic Science and Engineering Jilin University Changchun 130012 China; ^2^ The State Key Laboratory of Automobile Materials (Ministry of Education) School of Materials Science and Engineering Jilin University Changchun 130022 China; ^3^ International Center of Future Science Jilin University Changchun 130012 China

**Keywords:** alk‐Ti_3_C_2_T_x_, contact barrier difference regulation, motion object recognition, noncontact sensing, self‐powered pressure sensor

## Abstract

Wearable self‐powered pressure sensors present tremendous potential for object recognition. However, the fluctuated approach speed and distance may compromise the device output, thus affecting the recognition accuracy. Herein, a wearable self‐powered pressure sensor with high sensitivity (1.48 V kPa^−1^), high output (130.5V), and high permeability (259.98 mm s^−1^) are developed where the contact barrier difference and triboelectric charge density of the triboelectric layer surface are dynamically regulated by modulating the surface groups resulting in a lower dielectric loss and higher output. The sensor leverages the polarity difference between the target object and the sensitive material to generate electrical signals at different speeds and distances through an electrostatic induction mechanism. With the assistance of the Transformer model with a self‐attention mechanism, an average recognition accuracy of 94.3% is achieved by acquiring sensing signals at different speeds and distances. Simulating the ability of human vision, enables visually impaired people to acclimate to their surroundings more easily and independently and provides a critical advancement in the assistance of the blind with daily life.

## Graphical abstract

The contact barrier difference is dynamically regulated by modulating the surface groups and introducing a dielectric trapping failure layer, contributing to high‐performance noncontact pressure sensors. Furthermore, this study eliminated the interference of object recognition accuracy caused by different approach speeds and distances. This achievement brings significant advances to improve the daily lives of blind people.

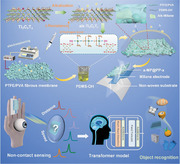

## 1 Introduction

With the recent advancements of the Internet of Things and artificial intelligence technology, object recognition has been widely used in various fields, such as healthcare,^[^ ^1^ ^]^ intelligent robots,^[^ ^2^ ^]^ and movement detection.^[^ ^3^ ^]^ Particularly, wearable pressure sensors with high sensitivity, stability, and interference resistance have been extensively investigated for tactile sensing.^[^ ^4^ ^]^ It can perceive different stimuli simultaneously and recognize contact objects through the electrical signal output generated by the amount of pressure, the force deformation, as well as the distribution of the pressure suffered. This process effectively and precisely translates object information into discernible electrical signals that can be used for additional classification and identification.

As an emerging pressure sensor technology, triboelectric pressure sensors (TPSs), based on triboelectrification and electrostatic induction, are considered promising wearable sensors for object recognition because of their noncontact sensing and low energy consumption.^[^ ^5^ ^]^ The object recognition process is based on the grip strength recognition technology by generating recognizable electrical signals associated with the difference in softness.^[^ ^6^ ^]^ By analyzing the relationship between force and displacement during the contact process, mechanical characteristics, such as Young's modulus of the object, can be quantified for further identification.^[^ ^7^ ^]^ Moreover, species recognition based on the different electron affinity between the sensor and the objects is an alternative highly efficient, and accurate method.^[^ ^8^ ^]^ This process can yield electrical signals through both contact and noncontact sensing, as specific triboelectric characteristics arise when sensors approach different objects due to differences in electron affinity. The object type and surface roughness can be accurately recognized by evaluating information on the specific triboelectric properties.^[^ ^9^ ^]^ When these sensors are attached to the human body or a robotic arm, they can momentarily generate electrical signals and recognize the objects.^[^ ^10^ ^]^ Thus, for triboelectric pressure sensors in complicated conditions, it proves a challenge to maintain a constant approach speed and distance between the wearable sensors and the objects.^[^ ^11^ ^]^ The elimination of speed and distance impacts on recognition accuracy is therefore essential. The sensing signals of an object at different speeds and distances need to be dynamically adjusted by fabricating TPSs with high sensitivity to acquire accurate signals and adopting an algorithmic model with an adaptive learning mechanism.

**Figure 1:**
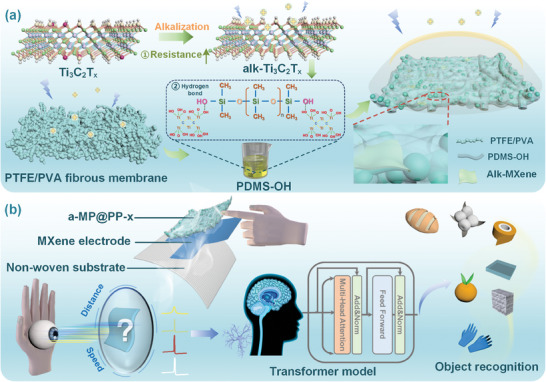
a) Schematic design diagram of the alk‐Ti_3_C_2_T_x_ MXene/PDMS@PTFE/PVA regulating contact barrier difference. b) The a‐MP@PP‐x pressure sensor with high air permeability and excellent output performance for motion object recognition assisted by the Transformer model.

The output performance of triboelectric pressure sensors is primarily determined by the dielectric effect and the charge capture effect. Therefore, it is possible to dynamically regulate the potential barriers on the surface of the triboelectric layer by enhancing the dielectric effect and avoiding the charge‐trapping effect. Ti_3_C_2_T_x_ MXene, an inorganic nanomaterial with a high dielectric constant, can strengthen the dielectric effect and boost capacitance to regulate the triboelectric charge density.^[^ ^12^ ^]^ The conductivity can be further decreased by the modulation of Ti_3_C_2_T_x_ surface groups. The regulation of the terminal group contributed to the increase of resistance and the dispersion of alk‐Ti_3_C_2_T_x_, which hinders the formation of internal conductive pathways and reduces the dielectric loss. Besides, a charge‐capture failure layer with poor surface conductivity is critically important to prevent the entrapment of charges generated by air ionization. It diminishes the influence of the charge trapping effect on output performance and increases the contact barrier difference between the triboelectric layers, as well as preserving the fiber structure and keeping the air permeability of the triboelectric layer for wearable comfort (**Figure** **1**a).^[^ ^13^ ^]^

Herein, a wearable comfortable self‐powered pressure sensor with high output performance and high permeability (259.98 mm s^−1^) is designed. It exhibits an ultralow detection limit (0.94 Pa), high stability (over 10 000 cycles), and high sensitivity (1.48 V·kPa^−1^). An innovative approach for recognizing moving objects that exclude the interference of moving speed and distance has been proposed by investigating the influence factors of the output performance through simulation. To improve the detection of moving objects, a Transformer model is used for training the response signals under different moving speeds and distances (Figure 1b). It may assist visual perception by tactile sensing and has a widespread application in accessible travel for blind people.

## 2 Results and Discussion

### 2.1 Characterization of a‐MP@PP‐x Membrane

**Figure 2:**
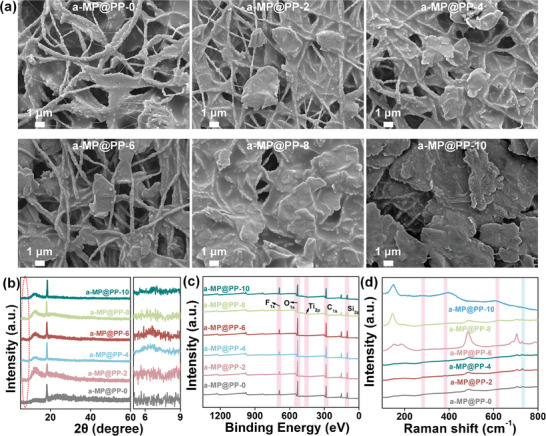
Characterization of a‐MP@PP‐x. a) SEM images of a‐MP@PP‐x. b) XRD patterns c) XPS spectra and d) Raman spectra of a‐MP@PP‐x.

The fabrication process of alk‐Ti_3_C_2_T_x_/PDMS@PTFE/PVA (a‐MP@PP‐x) is displayed in Figure S1 (Supporting Information). Alk‐Ti_3_C_2_T_x_ MXene was prepared by alkalinization of Ti_3_C_2_T_x_ to remove the fluorine atom and gain the hydroxyl group. The morphology of Ti_3_C_2_T_x_ and alk‐Ti_3_C_2_T_x_ was characterized by TEM and SEM. Ti_3_C_2_T_x_ and alk‐Ti_3_C_2_T_x_ all displayed a lamellar structure and the selected area diffraction (SAED) pattern illustrated the same crystalline phase (Figures S2 and S3, Supporting Information). The distance of the lattice plane was expanded from 0.17 to 0.28 nm after alkalization possibly attributed to intercalation of Na^+^.^[^ ^14^ ^]^ The morphology of PTFE/PVA fibers was characterized by SEM and TEM, respectively, as demonstrated in Figure S4 (Supporting Information). The electrospinning PTFE/PVA displayed a necklace‐like structure before or after the crosslink (Figure S4b,c, Supporting Information). **Figure** **2**a illustrates the evolution of a‐MP@PP‐x membranes with the increase of alk‐Ti_3_C_2_T_x_ concentrations. The alk‐Ti_3_C_2_T_x_ MXene/PDMS was homogeneously coated on the surface of PTFE/PVA with the alk‐Ti_3_C_2_T_x_ MXene increased from 0% to 8%. When the alk‐Ti_3_C_2_T_x_ MXene concentration increased to 10%, the alk‐Ti_3_C_2_T_x_ MXene gathered on the fiber surface due to strong hydrogen bonding, and the fibrous structure was hard to observe (Figure S5, Supporting Information) and Ti element content on the surface was dramatically increased (Figure S6, Supporting Information). The images of the polarization microscope further demonstrated the structural evolution of a‐MP@PP‐x with alk‐Ti_3_C_2_T_x_ MXene concentration (Figure S7, Supporting Information).

The characterization structure of a‐MP@PP‐x was accomplished by XRD, Raman, and XPS. The XRD patterns of Ti_3_C_2_T_x_ and alk‐Ti_3_C_2_T_x_ MXene are shown in Figure S8a (Supporting Information). The (002) peak of Ti_3_C_2_T_x_ became strong and was shifted to the low‐angle direction after the alkalization, indicating that the spacing between the layers of alk‐Ti_3_C_2_T_x_ MXene became larger after alkalization, which can also be observed from the high‐resolution lattice diffraction patterns in TEM, which also proves the interaction of Na^+^.^[^ ^15^ ^]^ Moreover, the (004) peak was weakened and the (006) peak was slightly strengthened, which was consistent with the previous articles.^[^ ^16^ ^]^ As shown in Figure 2b, with the addition of alk‐Ti_3_C_2_T_x_, the (002) characteristic peak attributed to alk‐Ti_3_C_2_T_x_ became more apparent and shifted to a low‐angle direction, which illustrated that the mix of PDMS and alk‐Ti_3_C_2_T_x_ contributed to the dispersion of alk‐Ti_3_C_2_T_x_. To conduct further analysis of the chemical states and bonding configurations, the XPS patterns of Ti_3_C_2_T_x_, alk‐Ti_3_C_2_T_x,_ and a‐MP@PP‐x were tested. From the XPS survey spectra in Figure S8c (Supporting Information), compared with Ti_3_C_2_T_x_, the intensity of the F_1s_ peak of alk‐Ti_3_C_2_T_x_ decreased, the intensity of the O_1s_ peak increased, and a new Na_1s_ peak appeared, confirming that the Na^+^ intercalation. Based on the XPS spectra of Ti_2p_ and SEM‐EDS, the atomic number of O and F atoms terminals was calculated that the oxygen‐fluorine atom number ratio ([O]/[F]) was dramatically increased after alkalization (Figures S3,S8b, and Table S1, Supporting Information). From the XPS survey of a‐MP@PP‐x, the intensity of Ti_2p_ was increased with the increase of alk‐Ti_3_C_2_T_x_ concentration, and due to the low addition concentration of alk‐Ti_3_C_2_T_x_, the intensity of Ti_2p_ exhibited weak (Figure 2c; Figure S9, Supporting Information). The emerging Raman peak at 190 cm^−1^ of alk‐Ti_3_C_2_T_x_, is relevant to the displacement of Na^+^ along the c‐axis or the E_g_ vibrations of Ti‐O due to partial oxidation during alkalization^[^ ^17^ ^]^ (Figure S8d, Supporting Information). As shown in Figure 2d, the peaks at 283, 384, and 616 cm^−1^ assigned to the E_g_ group vibrations of the in‐plane shearing modes of Ti, C, and surface terminal atoms were demonstrated from a‐MP@PP‐2 to a‐MP@PP‐10.^[^ ^18^ ^]^ There was no peak belonging to C‐F symmetric stretching of PTFE at 730 cm^−1^ shown in a‐MP@PP‐10 due to the aggregation of alk‐Ti_3_C_2_T_x_ on the PTFE fiber surface.^[^ ^19^ ^]^

### 2.2 The Mechanism of a‐MP@PP‐x Self‐Powered Pressure Sensors

**Figure 3:**
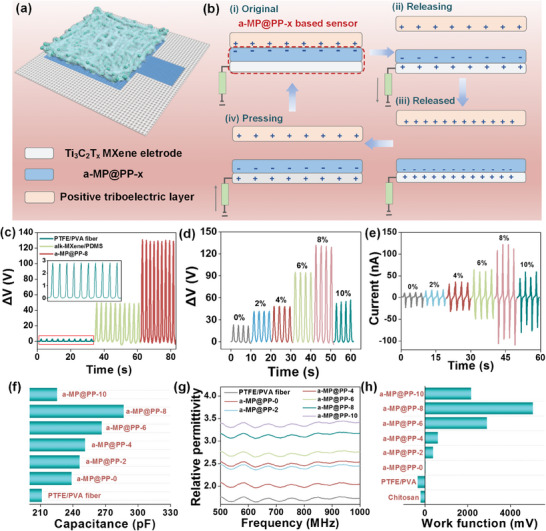
Performance optimization and mechanism analysis of a‐MP@PP‐8 pressure sensor. Schematic diagram of a) the device and b) the working principle of the device. c) The voltage response under 159.15 kPa pressure of PTFE/PVA fiber, alk‐Ti_3_C_2_T_x_/PDMS, and a‐MP@PP‐x pressure sensor. d)The voltage response and e) current of a‐MP@PP‐x with different alk‐Ti_3_C_2_T_x_ concentrations under 159.15 kPa. f) The capacitance and g) relative permittivity of PTFE/PVA and a‐MP@PP‐x. h) The work function of a‐MP@PP‐x, PTFE/PVA, and chitosan.

The configuration of the triboelectric pressure sensors crafted from the ultra‐high breathability a‐MP@PP‐x membrane is demonstrated in **Figure** **3**a. The a‐MP@PP‐x was employed as a negative triboelectric layer (Figure S10, Supporting Information), while chitosan aerogel as a positive triboelectric layer. The a‐MP@PP‐x triboelectric layer was employed to produce a transfer charge due to the difference in the electrons' bounding ability of contacting surfaces resulting in a potential difference. Ti_3_C_2_T_x_ MXene, as an electrode layer coated by screen printing on a PET non‐woven substrate with an ultralow resistance of 7.5 Ω (Figure S11, Supporting Information), served to transfer the induced charge generated by the triboelectric layer to flow through an external circuit and excellent conductivity provided efficient charge transfer.

Figure 3b demonstrates the processes of the contact charging and electrostatic induction of the pressure sensor in the single electrode mode. Initially, the chitosan positive layer was contacted with a‐MP@PP and was in an electrostatic balance state, where positive charges were collected on the chitosan surface and negative charges were collected on the a‐MP@PP‐x surface, and there was no current passing through the electrode layer. After separation, potential differences occurred on the surface between the two triboelectric layers. It induced the generation of an induced charge that free electrons flowed to the ground terminal through the electrode layer until the electrostatic balance state. Upon approaching, the electrostatic balance between the surfaces of the two triboelectric layers was disrupted again, and free electrons flowed through the electrode layer from the ground terminal. Subsequently, with the continuous contact/separation processed, the consistent alternating electrical signal was developed. During this process, the output voltage was primarily determined by the difference between the work function of the positive and negative triboelectric layers. The output voltage could be summarized as:^[^ ^20^ ^]^

(1) $$V=\frac{\left(\sigma_{0}-\Delta \sigma \right)\cdot d}{\varepsilon_{0}}-\frac{\Delta \sigma \cdot h}{\varepsilon_{0}\cdot \varepsilon_{r}}$$

where σ_0_ and Δσ represent the triboelectric charge density on the a‐MP@PP‐x and transferred charge density on the Ti_3_C_2_T_x_ MXene electrode, ε_0,_ and ε_r_ represent the vacuum permittivity and relative permittivity of the a‐MP@PP‐x, d is of two contact layers, h represents the thickness of a‐MP@PP‐x. Besides the structural factors such as the triboelectric layer thickness and the motion parameters such as distance and frequency, the main factors that determined the output voltage were permittivity and triboelectric charge density. The triboelectric charge density was associated with capacitance.^[^ ^21^ ^]^

(2) $$C_{max}=\varepsilon_{0}\cdot S\cdot \frac{\varepsilon_{r}}{h}$$

where S is the effective electrode area.

For a visual comparison of the sensor's voltage response with different alk‐Ti_3_C_2_T_x_ concentrations, and under different load pressures, motion speeds, and distances on the output voltage of the TPS, the voltage response of the sensor (ΔV) is defined as the difference between the output voltage under pressure loading and that in the unloaded state. To illustrate the contribution of alk‐Ti_3_C_2_T_x_ MXene and PDMS‐OH, the voltage of PTFE/PVA and alk‐Ti_3_C_2_T_x_ MXene‐based pressure sensors were measured. Compared with the PTFE/PVA and alk‐Ti_3_C_2_T_x_/PDMS‐based sensor, the voltage response of the a‐MP@PP pressure sensor was improved by ≈46 and 3 fold, respectively (Figure 3c), which was associated with dielectric effect and charge capture effect. PTFE fibers demonstrated a charge‐trapping effect due to internal defects, and the reverse charge produced by air ionization was caged in the defective state, resulting in a low voltage response. Consequently, the injection of 2D nanomaterials alk‐Ti_3_C_2_T_x_ with high permittivity enhanced the capacitance and permittivity of the triboelectric layer, leading to an increase in the voltage response. PDMS‐OH provided a charge‐trapping failure layer as a protective barrier outside PTFE fibers and alk‐Ti_3_C_2_T_x_, eliminating the charge‐trapping effect.^[^ ^22^ ^]^ Compared with Ti_3_C_2_T_x_, the conductivity of alk‐Ti_3_C_2_T_x_ decreased and the distance between nanosheets increased due to uniform dispersion in PDMS‐OH, leading to conductive pathways reduction of the dielectric layer. Thus, alk‐Ti_3_C_2_T_x_/PDMS@PTFE/PVA TPS exhibited lower dielectric loss and higher voltage response (Figure S12, Supporting Information).

The concentration of alk‐Ti_3_C_2_T_x_ also influenced the voltage response of the device. During the operation process, the negative triboelectric layer served as an energy harvesting and energy storage device and it was represented as a capacitor, where the addition of alk‐Ti_3_C_2_T_x_ corresponded to the accumulation of multiple micro capacitors.^[^ ^23^ ^]^ The overall capacitance was increased compared with a‐MP@PP‐0 (Figure 3f). As the concentration of alk‐Ti_3_C_2_T_x_ increased, the permittivity and the capacitance were enhanced, which contributed to the ability of energy storage^[^ ^24^ ^]^ (Figure 3g). Thus, the voltage response and current progressively enhanced with the concentration increased from 0% to 8% (Figure 3d,e). With the alk‐Ti_3_C_2_T_x_ concentration continued to increase, the distance between alk‐Ti_3_C_2_T_x_ nanosheets grew closer and more conductive pathways were formed within a‐MP@PP‐x, which led to the space charge conduction and enhancement of dielectric loss^[^ ^25^ ^]^ (Figure S13, Supporting Information). The aggregation of excessive alk‐Ti_3_C_2_T_x_ provoked a tunnel effect leading to the destruction of micro capacitor. Moreover, as the concentration of alk‐Ti_3_C_2_T_x_ reached 10%, PDMS‐OH charge trapping failure layer failed to cover all high dielectric particles, and since the irregularly arranged crystal defects on the surface of alk‐Ti_3_C_2_T_x_, it suffered a charge trapping effect leading to an increase in dielectric loss as well. These all consequently contributed to a decrease in the contact barrier difference between a‐MP@PP‐x and the positive triboelectric layer and a decrease in output performances. (Figure 3h).

### 2.3 The Performance of a‐MP@PP‐8 Self‐Powered Pressure Sensors

**Figure 4:**
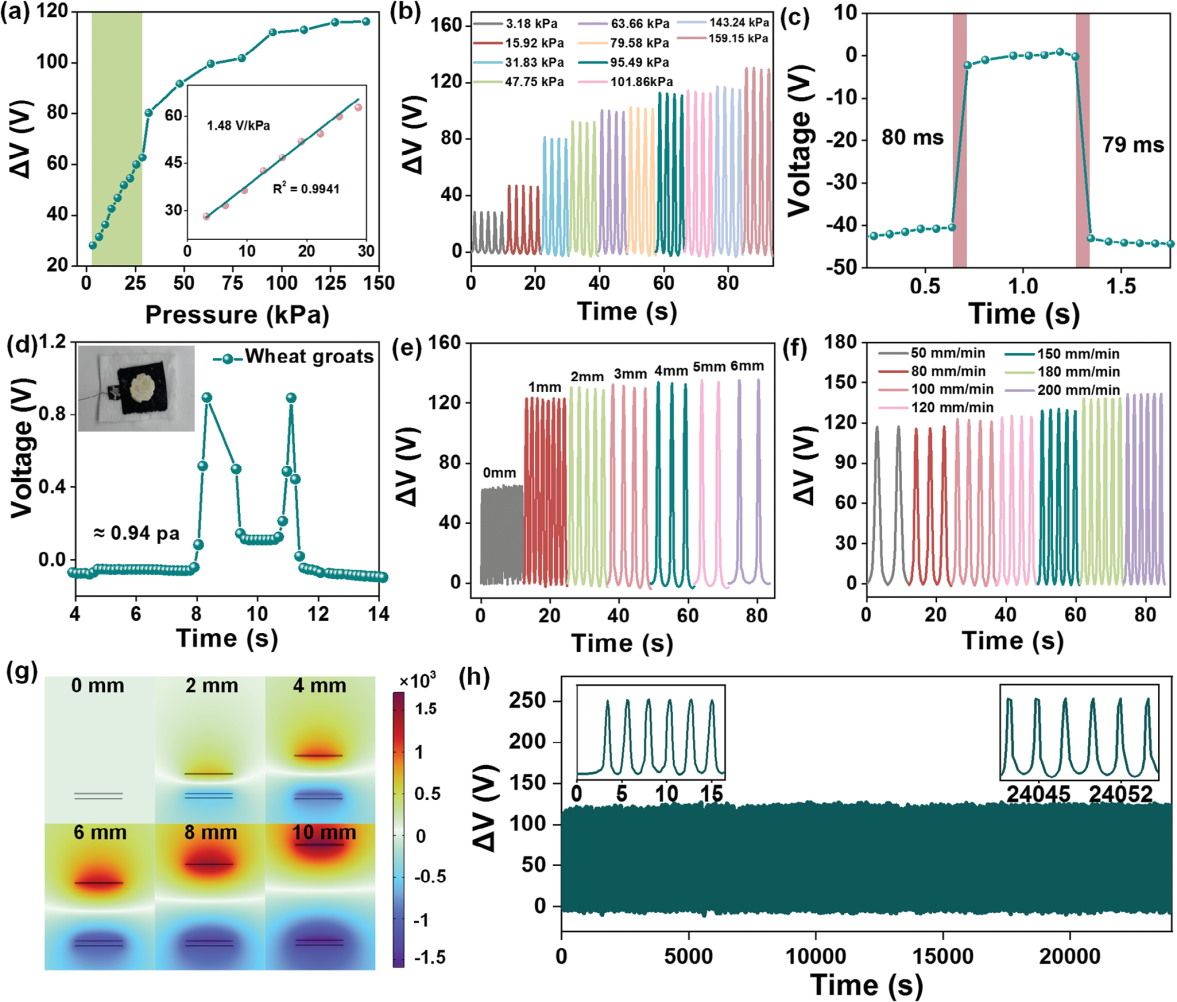
a) The voltage response‐pressure curves of a‐MP@PP‐8 pressure sensor (The inset illustrates the linear relationship between response and pressure within 30 kPa). b) The voltage response of a‐MP@PP‐8 pressure sensor under different pressures. c) The response and recovery time is under 15.92 kPa. d) The output voltage under wheat groats weighing 0.02g. The voltage response is under 159.15 kPa e) with different distances between two triboelectric layers and f) with different approaching speeds. g) The simulation of potential distribution at different distances. h) The durability test under 10 000 loading/unloading cycles at a force of 159.15 kPa, inset shows the enlarged curve for the first 6 cycles and last 6 cycles.

To assess the sensing performances, energy harvesting capabilities, and wearable comfort of the a‐MP@PP‐8 pressure sensor, further measurements are required. The sensitivity and the voltage response of the pressure sensors under different load pressures were investigated by varying the contact force at a constant distance of 2 mm between the two triboelectric layers and a constant approach speed of 120 mm min^−1^. The linear sensitivity of the sensor was as high as 1.48 V kPa^−1^ for the range of 0–30 kPa (R^2^ = 0.9941) (**Figure** **4**a; Figure S14, Supporting Information), which was superior to most wearable triboelectric pressure sensors (Table S2, Supporting Information). It demonstrated a distinguishable response signal for pressure that the voltage response was enhanced from 28.3 to 130.5 V when the load pressure was increased from 3.18 to 159.15 kPa (Figure 4b). The response/recovery time of the a‐MP@PP‐8 pressure sensor during pressure applied and removed as rapidly as 80 and 79 ms under 15.92 kPa was considerable as well (Figure 4c). The pressure sensor also demonstrated an ultralow detection limit of 0.94 Pa when a wheat groat weighing 0.02g and with ≈2.08 cm^−2^ contact area was loaded (Figure 4d). The a‐MP@PP‐8 pressure sensor was preferably employed as a sensor for object recognition and the output performance was measured at different dielectric distance and approach speeds. The voltage response rose proportionally with greater distances and faster approach speeds (Figure 4e,f). To visually express the distance effect on potential distributions, a COMSOL simulation was conducted. Figure 4g represents the potential with the change of dielectric distance from 0 to 10 mm. The results agreed with the experimental results discussed above (Figure 4g; Figure S15, Supporting Information). The mechanical properties of a‐MP@PP‐x TPS were stale and were not affected by the concentration of alk‐Ti_3_C_2_T_x_. Under 100 cycles of pressure loading and unloading, the strain of a‐MP@PP‐8 TPS did not increase, which indicated the structural stability after 100 cycles. This may be attributed to the electrical stability (Figure S16, Supporting Information). And the electrical stability was also a vitally important performance measure for wearable sensors. At a 159.15 kPa load pressure for 10 000 cycles, the a‐MP@PP‐8 pressure sensor remained stable with no degradation of the voltage response (Figure 4h). The pressure sensor also demonstrated good temperature stability. As shown in Figure S17 (Supporting Information), the voltage response of 3.18 kPa was stable after keeping various temperatures for 1 h.

**Figure 5:**
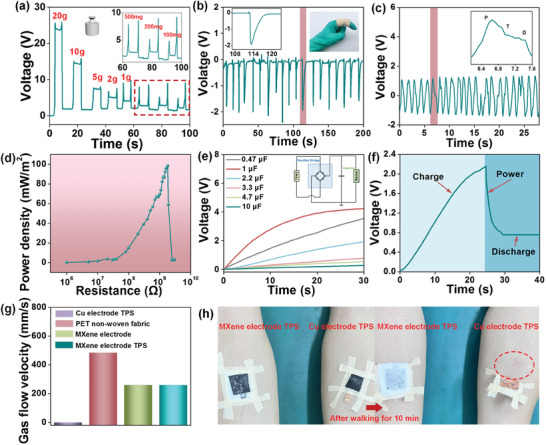
a) Voltage change of loading and unloading different weights from 100 mg to 20 g. b) Voltage change of finger bending. c) Real‐time recording of the wrist pulse waveform (The inset illustrates the enlarged view of the pulse vibration waveform). d) The power density of a‐MP@PP‐8 pressure sensor with different load resistance. e) Charging characteristic of the capacitors with different capacitance by a‐MP@PP‐8 pressure sensor (The inset illustrates the circuit diagram of the charging process). f) Charging and releasing curve of a 3.3 µF capacitor by a‐MP@PP‐8 pressure sensor when driving clocks. g) The air permeability of Cu electrode TPS, PET non‐woven substrate, Ti_3_C_2_T_x_ MXene electrode, and Ti_3_C_2_T_x_ MXene electrode TPS. h) Comparison of skin status after 10 min of walking with Cu electrode TPS (right) and Ti_3_C_2_T_x_ MXene electrodes TPS (left).

Furthermore, the ultrasensitive detection of different weights is possible, ranging from 100 mg to 20 g weights (**Figure** **5**a). The pressure sensors were also essential for the monitoring of human physiological signals that were attached to the finger joints and wrists of a 26‐year‐old female volunteer respectively. The flexion and extension of the finger joints can also be precisely monitored (Figure 5b). The wrist pulse has excellent periodicity and stability, beating regularly at 55 beats per min, and the three faint characteristic peaks of the human pulse can be distinctly distinguished: percussive (P), tidal (T), and diastolic (D) waves (Figure 5c). The a‐MP@PP‐8 pressure sensor illustrated the enormous promise of power supply for small electronic devices as well. The effect of load resistance on triboelectric pressure sensor power density should be evaluated at 159.15 kPa and for 120 mm/min with various resistances ranging from 1 MΩ to 3 GΩ. The power density of the pressure sensor was calculated using the formula P = U^2^/R/S, where U is voltage, R is external load resistance and S is the area of the triboelectric layer. The peak power density was 98.34 mW m^−2^ at a speed of 120 mm/min when the external load resistance reached 1.8 GΩ (Figure 5d; Figure S18, Supporting Information). It can be charged from 0.47 to 10 µF in 30 s via a rectifier bridge (Figure 5e). The pressure sensor could light the “MXene” and “TENG” LEDs easily (Movie S1, Supporting Information). Using a 3.3 µF capacitor to store the power, the clocks could operate for 4.8057 s (Figure 5f). The a‐MP@PP‐8 pressure sensor demonstrated an enhanced voltage response, a higher power density under low frequency, and higher air permeability (**Table** **1**). Additionally, during actual application, wearable electronic devices have to meet the requirements for wearable comfort such as air permeability and water contact angle, which were as high as 259.98 mm s and 126.88°, respectively (Figure 5g; Figure S19, Supporting Information). To further demonstrate the wearable comfort, the pressure sensor of a‐MP@PP‐8 with Ti_3_C_2_T_x_ electrode and with Cu electrode was attached to the skin surface of a 25‐year‐old volunteer. After 10 min of walking, the skin surface wearing the pressure sensor with Cu electrode became erythema and sweating heavily. The condition of the skin surface wearing that with Ti_3_C_2_T_x_ electrode did not change (Figure 5h).

**Table 1:** Comparison of the voltage response, power density, and air permeability between the prepared self‐powered pressure sensor in this article and previous articles.

Voltage response [V]	Power density [mW m^−2^]	Air permeability [mm s^−1^]	Reference
200	31		[26]
87.98 (1–5Hz)	38.64		[27]
50	76.85		[28]
<6	0.031		[29]
90	93	164 ± 5	[30]
7.5 (1.5Hz)		333	[31]
168 (3Hz)	1030	3.2	[32]
50 (1Hz)	46.1		[33]
114.5 (4Hz)	445	215	[34]
80 (2Hz)	11.5		[35]
22 (7Hz)		189	[36]
30 (4Hz)		152.47	[37]
76 (6Hz)	200.93		[38]
130.5 (0.5Hz)	98.34	259.98	This work

### 2.4 Deep Learning‐Assisted Motion Object Identification Based on a‐MP@PP‐8 Pressure Sensor

**Figure 6:**
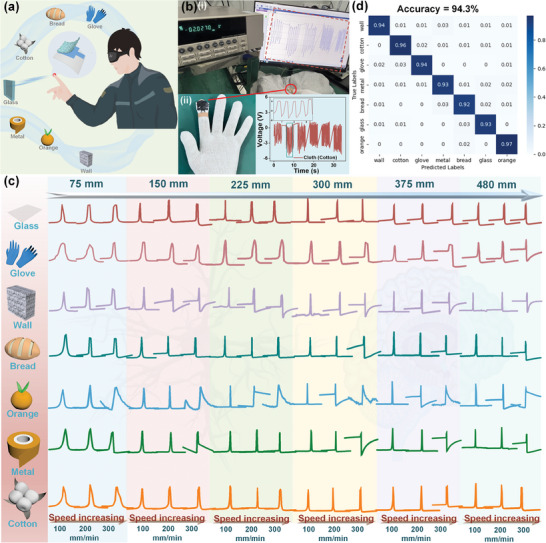
Motion object recognition is assisted by deep learning. a) Schematic of object recognition for the blind. b) The sensing signals acquisition by the wearable TPS. c) The sensing signals are acquired by a‐MP@PP‐8 pressure sensor at different speeds and different distances. d) The confusion matrix of the Transformer model for 7 objects at different speeds and different distances.

For the blind, a more autonomous lifestyle demands the ability to obstacle avoidance safely and efficiently. Noncontact tactile sensing technology is an effective strategy to replace human vision that overcomes the physical weakness of visual impairment (**Figure** **6**a). Thus, we have fabricated a noncontact wearable pressure sensor to acquire the specific electrostatic induction signals of different objects. The motion speed of the human and the distance from objects are dynamically varying, the voltage output as well as the potential distribution of the pressure sensors are also influenced by the speed and the distance between them (Figure 6b; Movie S2, Supporting Information). Hence, we controlled the speed and distance between the sensor and the object through the stepper motor to acquire the electrical signal, and the limiter ensured that the sensor and the object were exposed to only slight contact to avoid the interference of contact force. The positive triboelectric layer kept on changing including gloves, glass, metal, orange, bread, cotton, and wall. 1509 sensing signals for 7 objects were obtained by a‐MP@PP‐8 sensor with different approaching speeds (100, 200, and 300 mm min^−1^) and distances (75, 150, 225, 300, 375, 480 mm) between the sensor and objects (Figure 6c; Figure S20, Supporting Information).

Here, the transformer, a neural network model based on the self‐attention mechanism, was employed for accurately classifying these sensing signals. First, the time series signals and the belonging labels were constructed as a dataset. This dataset was divided equally into five equal parts for training and testing according to the cross‐validation to avoid overfitting the first part was used as a test set and the remaining four parts were used as a train set, in the first experiment. In the second experiment, the second part was employed as the test set and the remaining four parts were used as the train set, and so on for five experiments. Since this task was to classify objects, only the transformer's encoder was used. The model could be divided into five parts, first, the dataset was transformed in dimension to adapt the structure of the Transformer Encoder, and then the dimension transformation dataset was fed into the Transformer Encoder layer, the dropout of the Transformer Encoder layer was set to 0.1 to decrease the overfitting, and the number of the encoder layers was eight layers. The optimizer was Adam who could adaptively adapt the learning rate of each parameter. The next part contained a normalization layer that standardized the inputs to a distribution with mean 0 and variance 1 for improving the training of the model. The data was then restored to its original dimensions, ready for feeding into the fully connected layer. The last part was a fully connected layer, serving to classification and outputting seven results with an average accuracy of 94.3% (Figure 6d; Figure S21, Supporting Information). This noncontact sensor assisted by deep learning can make it crucial in accessible travel of the blind in the future.

## 3 Conclusion

A self‐powered pressure sensor with high output under a low frequency (130.5V) and high air permeability (259.98 mm s^−1^) is developed for movement object recognition. It is achieved by the dynamic regulation of surface contact barrier difference attributed to the high dielectric constant nanoparticles and polymer charge trapping failure layer. The self‐powered pressure sensor possessed an ultralow detection limit (0.94 Pa) and high sensitivity (1.48 V kPa^−1^). Meanwhile, a novel method for efficiently recognizing movement objects has been proposed by obtaining 1509 electrical signals at different running speeds and distances, for training the Transformer model. It can eliminate the interference of running speed and distances on the recognition accuracy, with the accuracy of cross‐validation as high as 94.3%. This high‐performance self‐powered pressure sensor may provide crucial guidance for human‐computer interaction in the future.

## 4 Experimental Section

### Preparation of Alk‐Ti_3_C_2_T_x_ MXene

Alk‐Ti_3_C_2_T_x_ MXene nanosheets were produced through a less aggressive etching and alkalization process. Initially, 1 g LiF (Aladdin, > 99.99%) was dissolved in 40 mL of 12 m HCl (Xilong Scientific Co., Ltd.) within a Teflon lining with vigorous stirring for 15 min at room temperature. Subsequently, 1 g Ti_3_AlC_2_ (Jilin 11 Technology Co., Ltd., China) was incrementally added to the mixed solution. The reaction took place at 45 °C in a water bath for 24 h. After the etching process, the obtained mixture underwent repeated centrifugation for 8 min at 8000 rpm until the pH of the supernatant stabilized between 6 and 7. The clay‐like mixture was then ultrasonically dispersed in an ice bath at a power of 350 W for 90 min and the Ti_3_C_2_T_x_ MXene was isolated through centrifugation at 10 000 rpm for 10 min.

Then 150 mL^−1^ 5 wt.% NaOH solution was slowly added into 120 mL^−1^ of 8 mg·mL^−1^ Ti_3_C_2_T_x_ solution with continuous magnetic stirring at room temperature for 180 min. The alk‐Ti_3_C_2_T_x_ was obtained after repeated washing and drying in a 60 °C vacuum oven for 24 h. The collected alk‐Ti_3_C_2_T_x_ powder was used for further device fabrication.

### Preparation of PTFE/PVA Fibrous Membrane

Polytetrafluoroethylene (PTFE)/polyvinyl alcohol (PVA, Aladdin, Mw ≈67000) fibrous membrane was fabricated by electrospinning. The electrospinning precursor was prepared by mixing 7 mL^−1^ 60 wt.% PTFE solution (DISP 30, DuPont) and 5 mL^−1^ 10 wt.% citric acid (CA, Aladdin)/PVA solution and placed in 5 mL^−1^ syringes controlled by programmed syringe pumps with 0.4 mL·h^−1^ feed rate. 14 kV high voltage was provided by a high voltage power supply with a nozzle‐to‐collector distance of 15 cm. During the electrospinning process, the ambient humidity and temperature were maintained at a constant level of 38 ± 2% and 20 °C, respectively. The obtained fibrous membrane was cross‐linked in an oven at 160 °C for 30 min.

### Preparation of Alk‐Ti_3_C_2_T_x_ MXene/PDMS@PTFE/PVA

Different weight of alk‐Ti_3_C_2_T_x_ was added into 15 mL^−1^ cyclohexane (Macklin, >99%) solution of hydroxyl‐terminated polydimethylsiloxane (PDMS‐OH, Shandong Xingchi Chemical Technology Co., Ltd., China) (containing 4 mL^−1^ of PDMS). After stirring for 10 h, the curing agent was added to the above solution and the obtained cross‐linked PTFE/PVA was immersed in the above solution coated evenly, and then immediately taken out to be cured in air. The obtained membrane was defined as alk‐Ti_3_C_2_T_x_ MXene/PDMS@PTFE/PVA (a‐MP@PP‐x), x represented the mass percentage of alk‐Ti_3_C_2_T_x_ in PDMS, x = 0, 2, 4, 6, 8, 10, respectively.

### The Fabrication of a‐MP@PP‐x Pressure Sensor

The Ti_3_C_2_T_x_ MXene non‐woven electrodes were prepared by screen printing. PET nonwoven fabric was ultrasonically washed in an ultrasonic cleaner for 30 min, dried, and used in the subsequent steps. 50 mg·mL^−1^ Ti_3_C_2_T_x_ MXene colloidal solution was employed as printing ink. The Ti_3_C_2_T_x_ ink was printed on the PET fabric through a screen printing plate with 300 mesh, which was dried in a vacuum oven at 40 °C for 2 h. Then the a‐MP@PP‐x membrane was cut into 2 cm × 2 cm, coated with PDMS at the edge which was adhered to the Ti_3_C_2_T_x_ MXene fabric electrodes. The pressure sensor was fabricated after 5 min of curing.

### Characterizations

The morphology of Ti_3_C_2_T_x_ MXene nanosheet, alk‐Ti_3_C_2_T_x_ nanosheet, PTFE/PVA fibrous membrane, and a‐MP@PP‐x (x = 0, 2, 4, 6, 8, 10) was observed by scanning electron microscopes (FESEM, JEOL JSM‐7900F) with an acceleration voltage of 5 kV. The morphology of Ti_3_C_2_T_x_ MXene, alk‐Ti_3_C_2_T_x_ nanosheet, and PTFE/PVA fibrous membrane was observed by transmission electron microscopy (TEM; JEM 2100 F) with an acceleration voltage of 200 kV. The crystalline structures were characterized by wide‐angle X‐ray diffraction (XRD, Rigaku D/Max 2550) in the 2θ range from 3° to 50° with Cu Kα radiation (*λ* = 1.5418 Å). The scanning rate was 5° min^−1^. The chemical composition of the alk‐Ti_3_C_2_T_x_/PDMS@PTFE/PVA surfaces was examined by X‐ray photoelectron spectroscopy (XPS, ESCALAB 250) with an X‐ray source (Al Kα hυ = 1486.6 eV). The Raman spectra from 100 to 800 cm^−1^ were analyzed using a 632 nm laser by a Raman confocal micro‐spectrometer (LabRAM HR Evolution, Horiba, France). The work function was measured by the Kelvin probe (KP 6500 Digital, Kelvin Probe McAllister Technical Services Co., Ltd.). The Relative permittivity and dielectric loss were measured by a vector network analyzer (Keysight P9375A) from 500 MHz to 1000 MHz. The air permeability of the fabric was determined by following the standard SNT 2558.12‐2016.

### Pressure‐Sensing Performances

The different pressure conditions were achieved by a universal testing machine (AGS‐X, Shimadzu, Kyoto, Japan). The short‐circuit current, output voltage, and signal acquisition for the deep‐learning model of the pressure sensor were measured by an electrometer (Keithley, Keithley 6514). A programmable linear motor (CL‐01A) was utilized to control the contact and separation between the pressure sensor and the testing object in the data acquisition process under different speeds and distances.

### Finite Element Simulation of the Electric Potential Distribution

The COMSOL Multiphysics software was utilized to conduct the finite element simulation. For simulating the electric potential distribution, the “Electrostatics” module, coupled with the “Stationary” study, was employed. First, we build the geometric model of the pressure sensor. The negative triboelectric layer, a‐MP@PP‐x was set to be 5 mm width and 0.01 mm height, and the MXne electrode with 5 mm width and 0.01 mm height was under the negative triboelectric layer. The positive triboelectric layer chitosan (5 mm × 0.1 mm) was set above the a‐MP@PP‐x. Moreover, the surface charge densities of the top surface of the negative triboelectric layer were set to be −8 × 10^−6^ Cm^−2^, and that of the bottle surface of the positive triboelectric layer was set to be 8 × 10^−6^ Cm^−2^. Subsequently, the boundary was designated as an infinite element domain, and the mesh was constructed on the models by the Free Triangular feature node. Ultimately, the computation was executed to acquire the distribution of the electric potential and the output voltage was the electric potential difference between the bottle negative triboelectric layer and the top electrode layer.

## Conflict of Interest

The authors declare no conflict of interest.

## Supporting information



Supporting Information

Supplemental Movie 1

Supplemental Movie 2

## Data Availability

The data that support the findings of this study are available from the corresponding author upon reasonable request.
