# Supplementary material for: Wearable Self‐Powered Pressure Sensors Based on alk‐Ti3C2Tx Regulating Contact Barrier Difference for Noncontact Motion Object Recognition
Source: Adv Sci (Weinh). 2025 Feb 7;12(13):2416504. doi: 10.1002/advs.202416504 (PMC11967761; doi:10.1002/advs.202416504)
Supplement: Supplementary file 1 — Supporting Information [file ADVS-12-2416504-s001.docx]

Supporting Information

Wearable self-powered pressure sensors based on alk-Ti_3_C_2_T_x_ regulating contact barrier difference for non-contact motion object recognition

*Yanan Xiao ^#1^, Qi Pu ^#1^, Chenxing Wang ^1^, Xiaoteng Jia ^1*^, Shixiang S**un ^1^, Quan Jin ^2^, Xiaolong Wang ^1^, Bin Wang ^1^, Peng Sun ^1, 3^, Fangmeng Liu ^1,3*^, Geyu Lu ^1, 3*^*

Y. X., Q. P., C. W., X. J., S. S., X. W., B. W., P. S., F. L., G. L.

^1^ State Key Laboratory of Integrated Optoelectronics, College of Electronic Science and Engineering, Jilin University, Changchun 130012, China.

1. J.

^2^ The State Key Laboratory of Automobile Materials (Ministry of Education), School of Materials Science and Engineering, Jilin University, Changchun 130022, China

P. S., F. L., G. L.

^3^ International Center of Future Science, Jilin University, Changchun 130012, China.

#: These authors contribute equally.

**Keywords:** alk-Ti_3_C_2_T_x_, self-powered pressure sensor, contact barrier difference regulation; non-contact sensing, motion object recognition


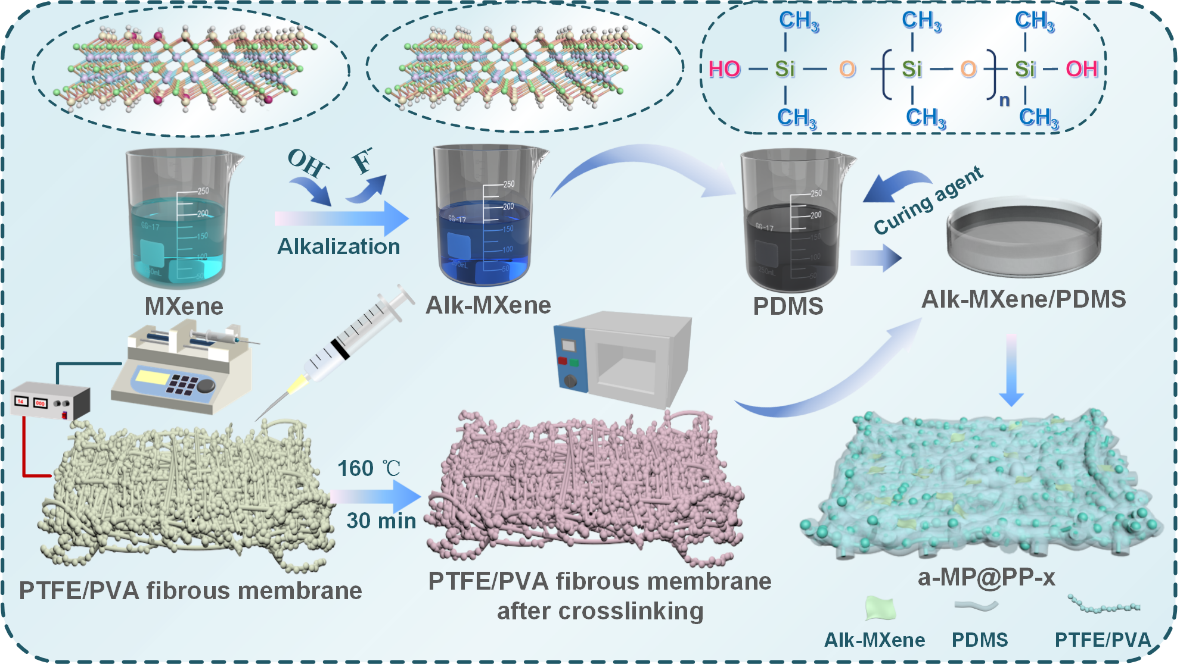


**Figure S1.** Schematic of the fabrication process of a-MP@PP-x.


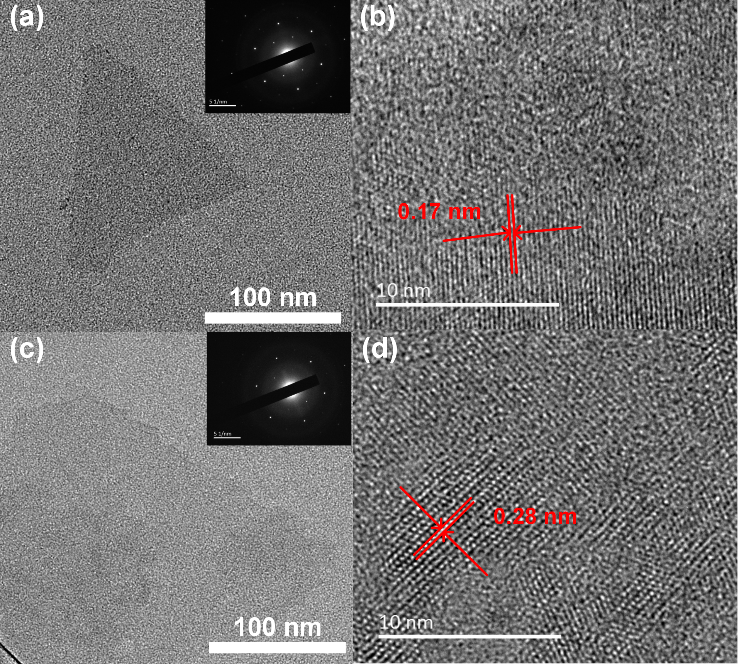


**Figure S2.** (a)TEM images and (b) HETEM image of Ti_3_C_2_T_x_ MXene nanosheet, and the inset was the SAED pattern. (c) TEM images and (d) HETEM image of alk-Ti_3_C_2_T_x_ MXene nanosheet, and the inset was the SAED pattern.


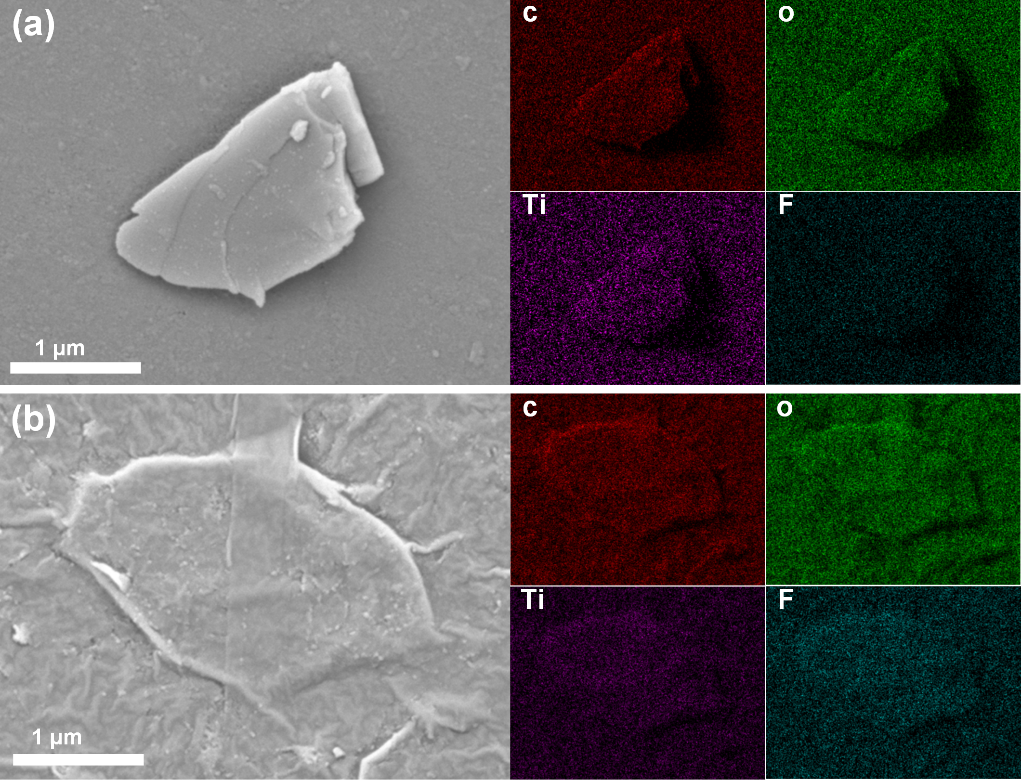


**Figure S3.** SEM and corresponding EDX elemental mapping images of (a) alk-Ti_3_C_2_T_x_ MXene, (b) Ti_3_C_2_T_x_ MXene.


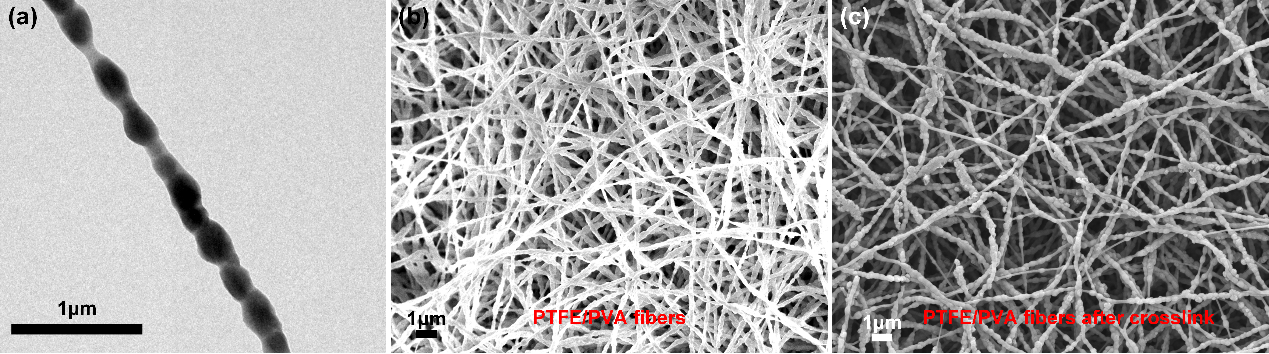
**Figure S4.** The morphology characterization of PTFE/PVA fibers. (a)TEM images. SEM image of PTFE/PVA fibers (b) before crosslink and (c) after crosslink.


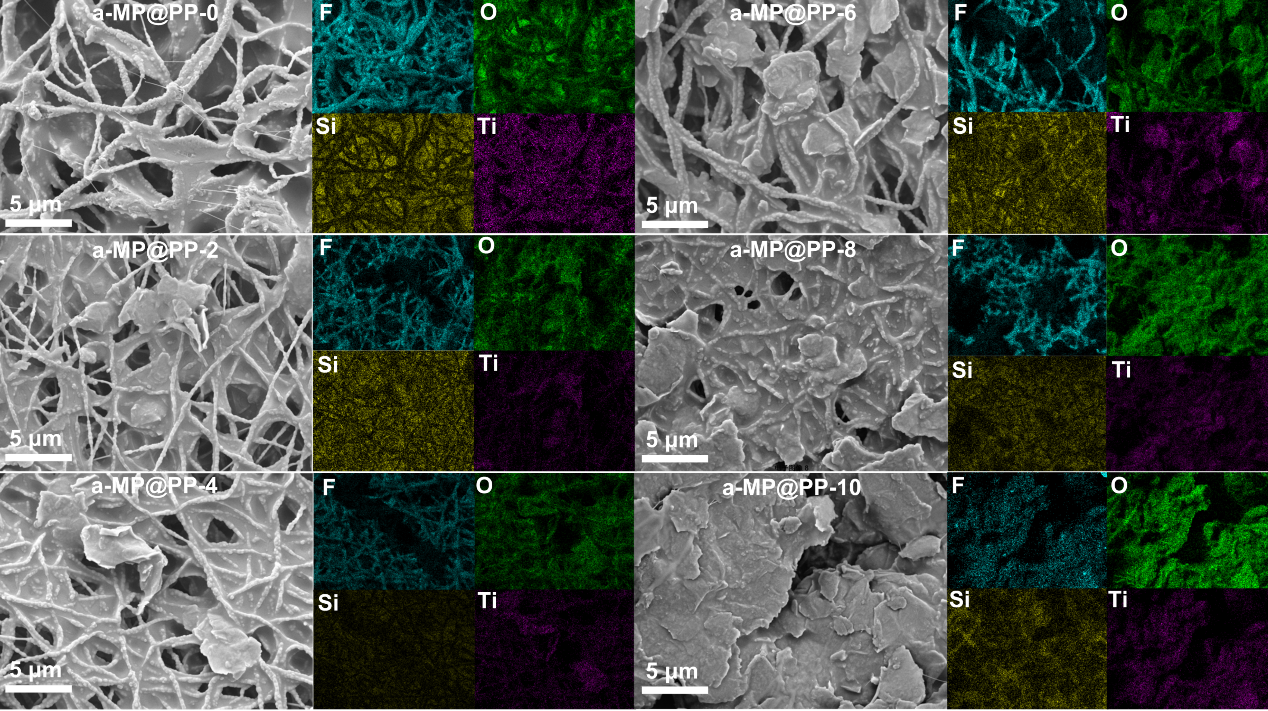


**Figure S5. S**EM and corresponding EDX elemental mapping images of a-MP@PP-x (x=0, 2, 4, 6, 8 and 10).


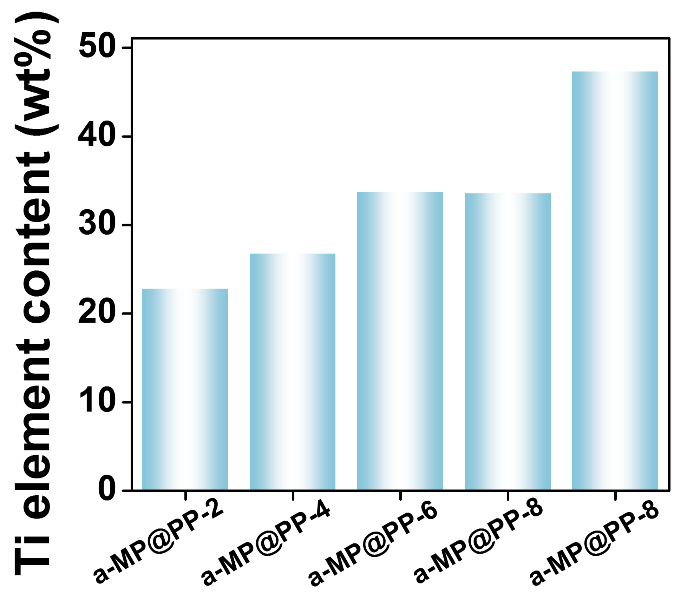


**Figure S6.** Ti element content of a-MP@PP-x tested by elemental mapping.


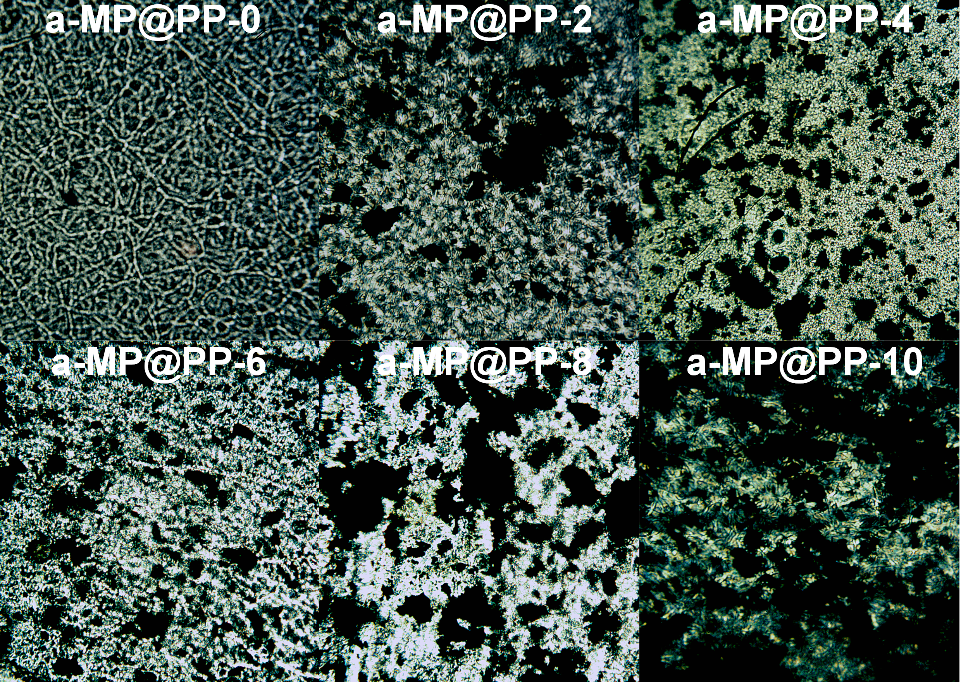


**Figure S7.** Polarization microscope images of a-MP@PP-x (x=0, 2, 4, 6, 8, 10).


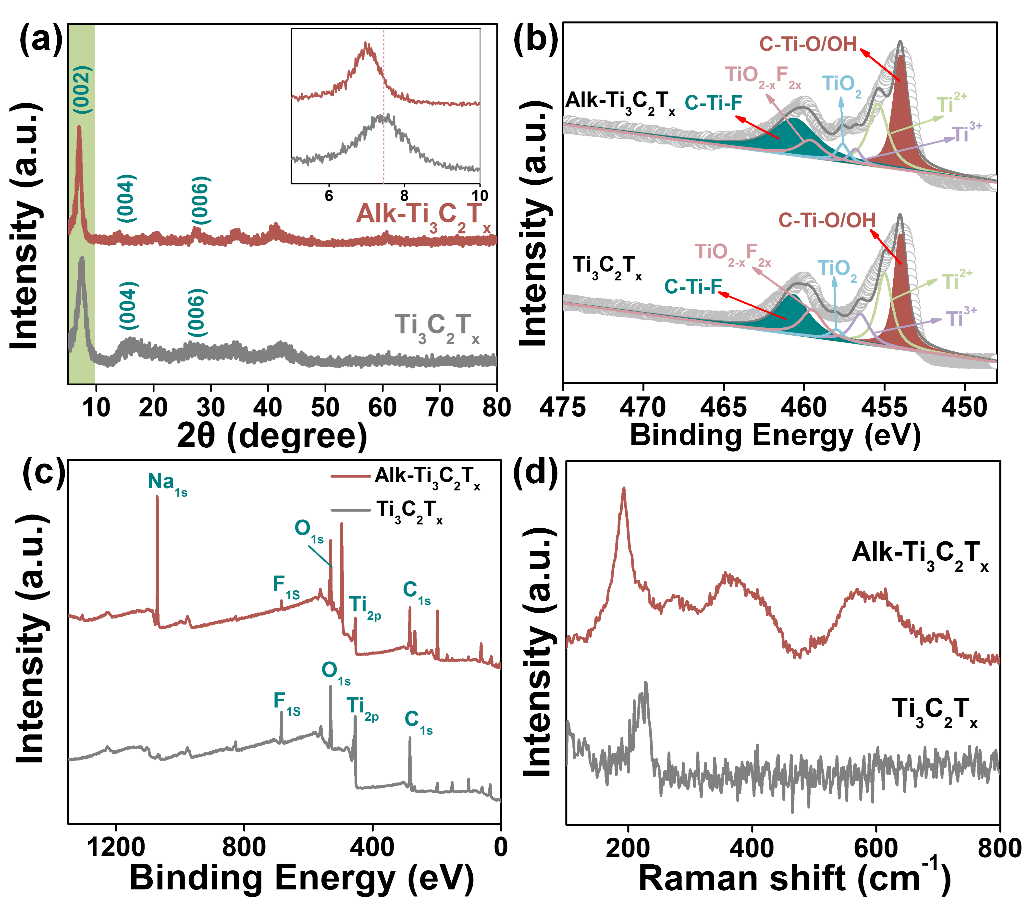


**Figure S8.** Characterization of Ti_3_C_2_T_x_ MXene and alk-Ti_3_C_2_T_x_ MXene. (a) XRD patterns. (b) Ti_2p_ XPS spectra. (c) XPS spectra. (d) Raman spectra.


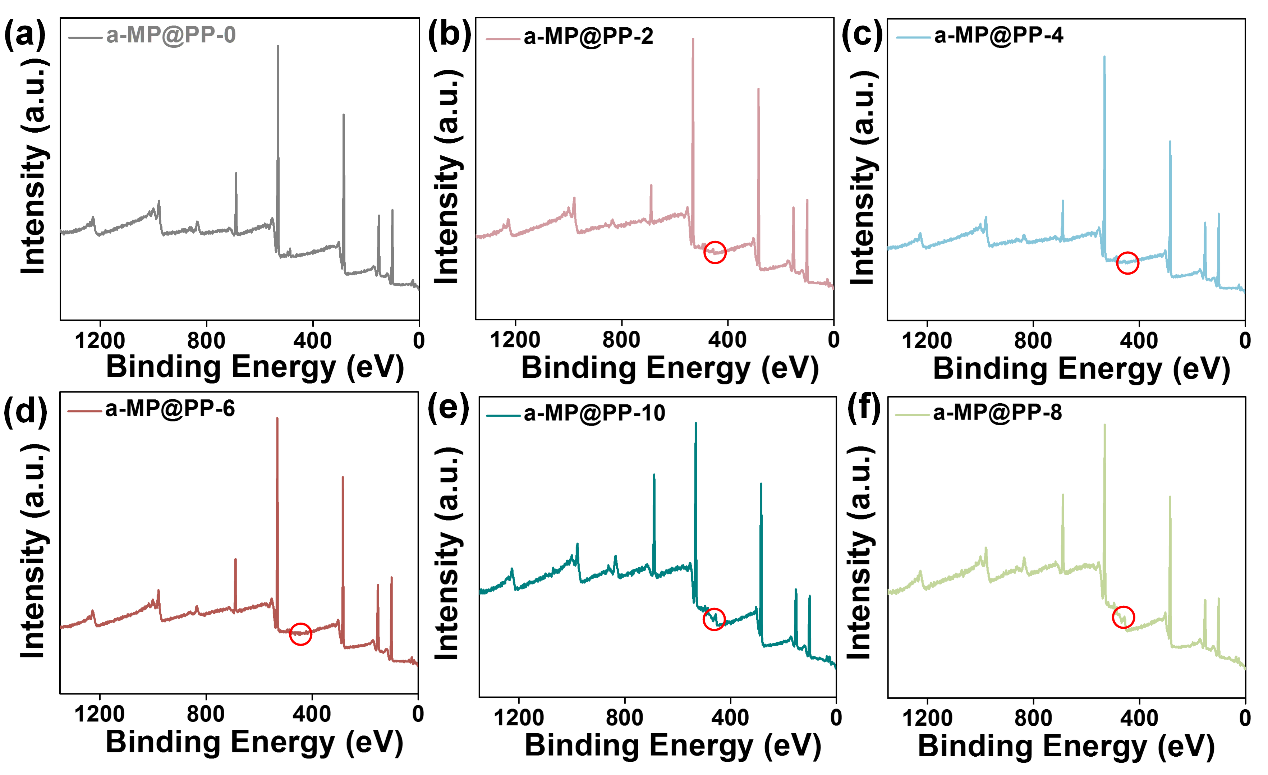


**Figure S9.** XPS spectra of a-MP@PP-x (x=0, 2, 4, 6, 8, 10).


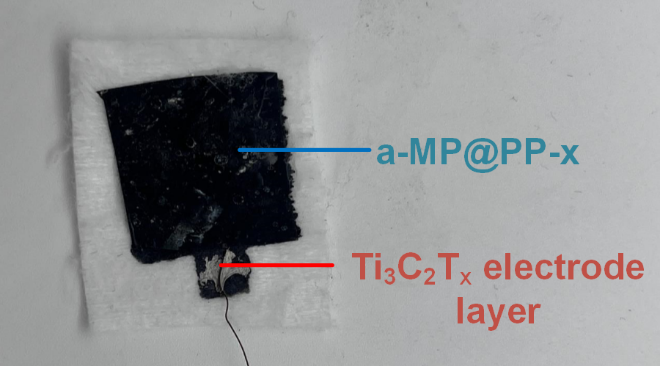


**Figure S10.** Photograph of a-MP@PP-x triboelectric pressure sensor.


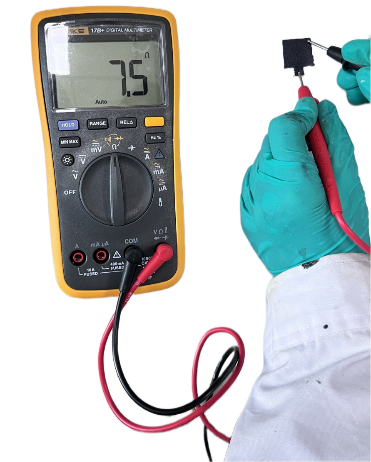


**Figure S11.** The resistance of Ti_3_C_2_T_x_ MXene electrode.


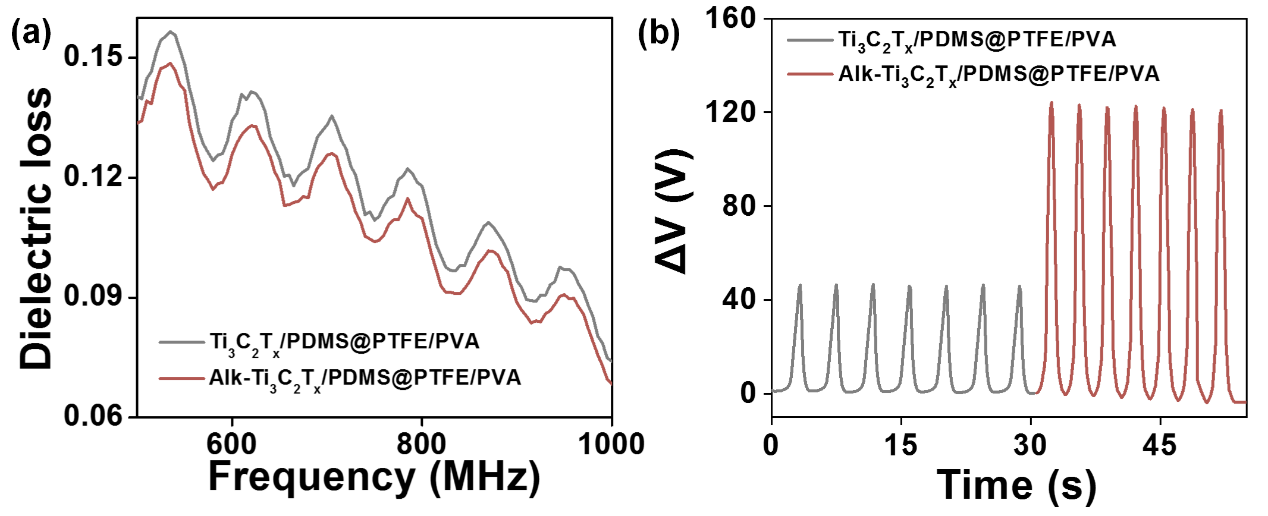


**Figure S12.** (a) The dielectric loss and (b) the output voltage of Ti_3_C_2_T_x_/PDMS@PTFE/PVA and alk-Ti_3_C_2_T_x_/PDMS@PTFE/PVA TPS.


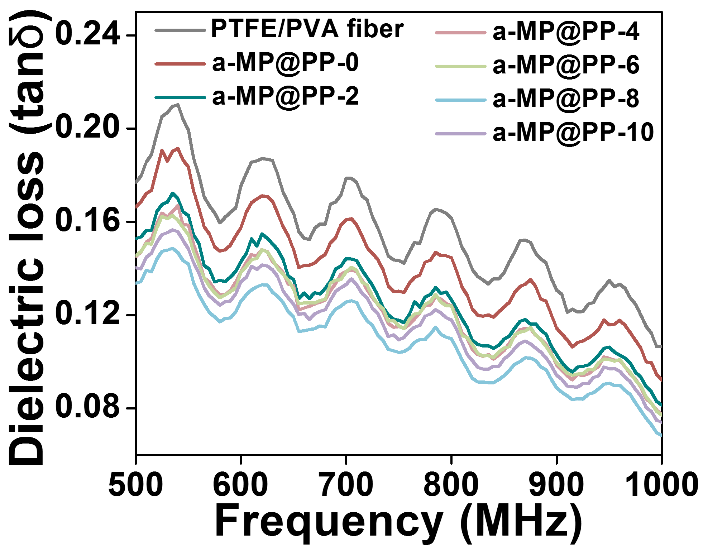


**Figure S13.** The dielectric loss of a-MP@PP-x (x=0, 2, 4, 6, 8, 10).


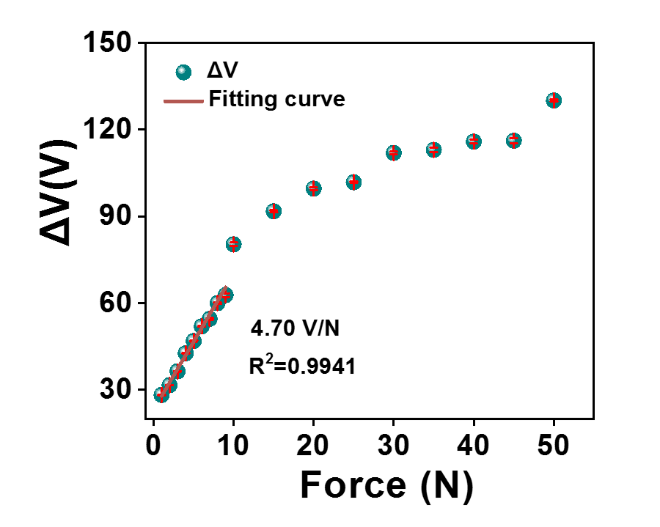


**Figure S14.** The sensitivity of a-MP@PP-8 based pressure sensor.


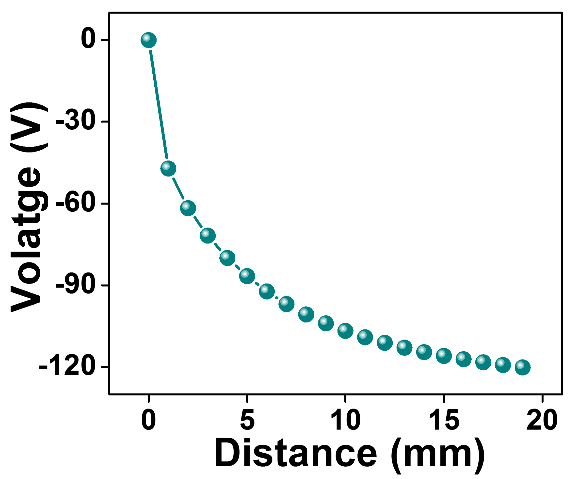


**Figure S15.** The simulation of the output voltage at different distances.


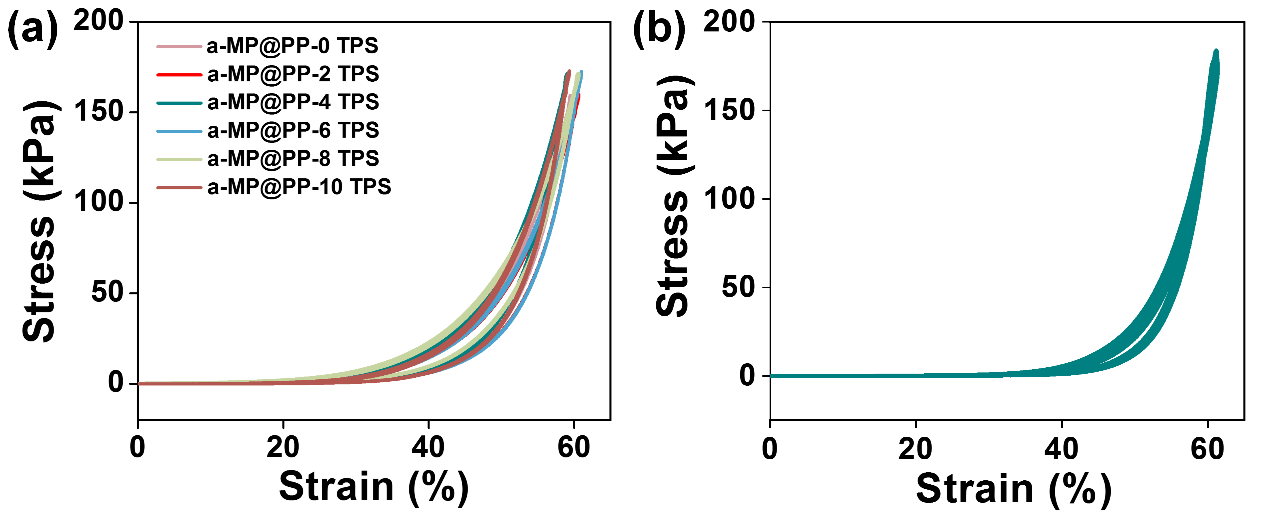


**Figure S16.** Stress-strain curves of a-MP@PP-x TPS (a) during pressure loading and unloading and (b) under 100 cycles of pressure loading and unloading.


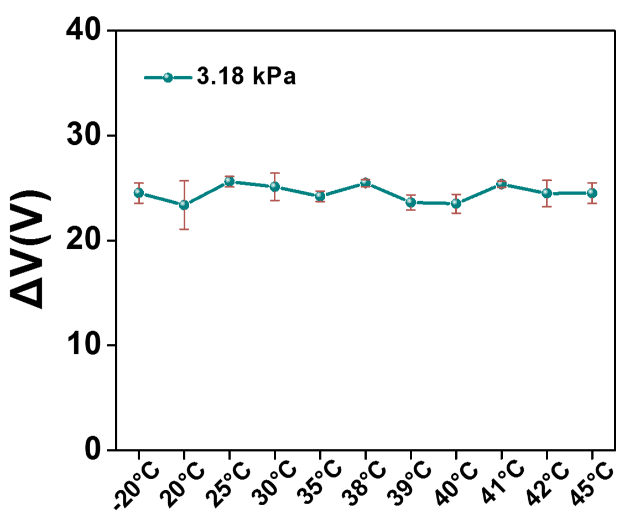


**Figure S17**. The voltage response of a-MP@PP-8 pressure sensor under different temperature for 1 hour.


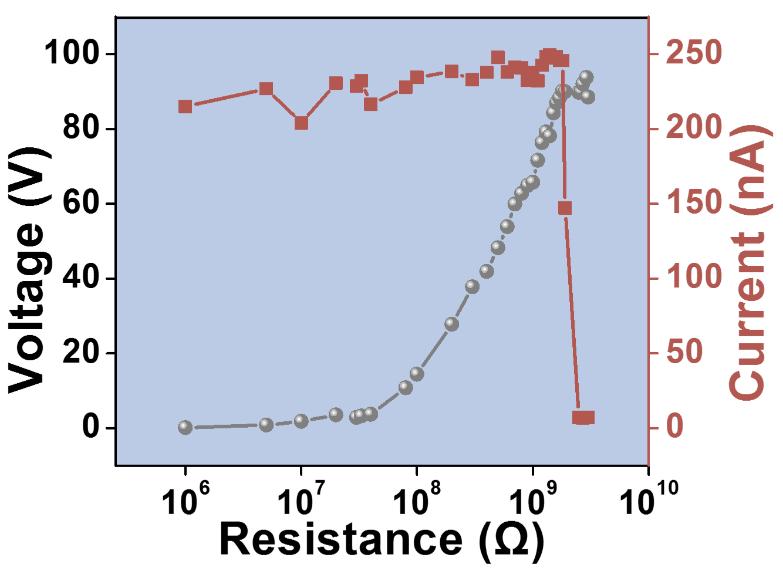


**Figure S18.** The voltage and current of a-MP@PP-8 based TENG with different load resistance.


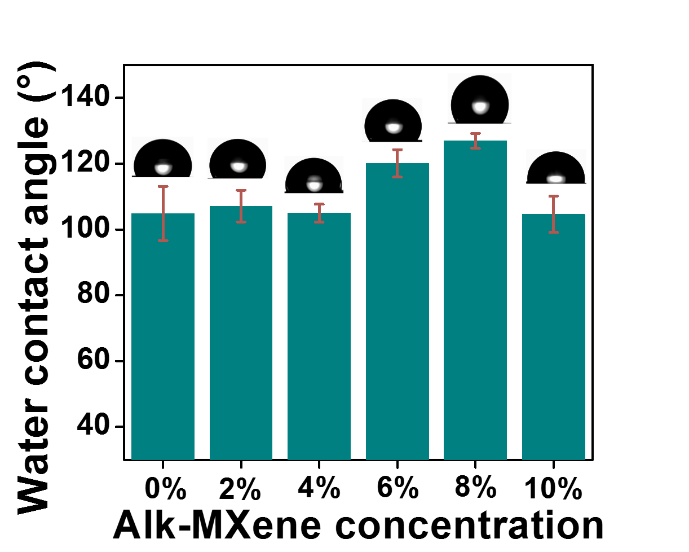


**Figure S19.** The water contact angle of a-MP@PP-x (x=0, 2, 4, 6, 8, 10).


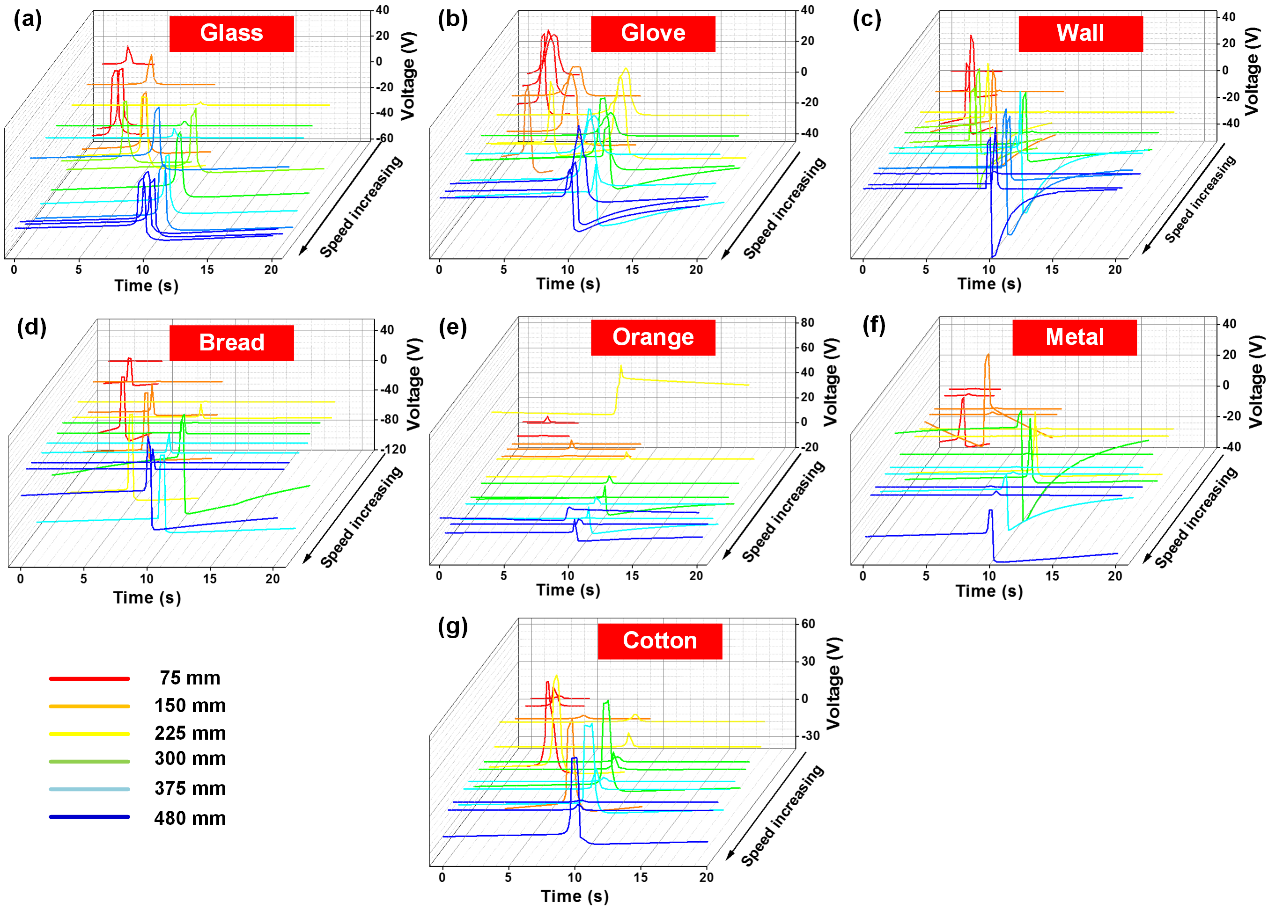


**Figure S20.** The real-time sensing signals for different objects are acquired by a-MP@PP-8 pressure sensor at different speeds and different distances (a) glass, (b) glove, (c) wall, (d) bread, (e) orange, (f) metal and (g) cotton.


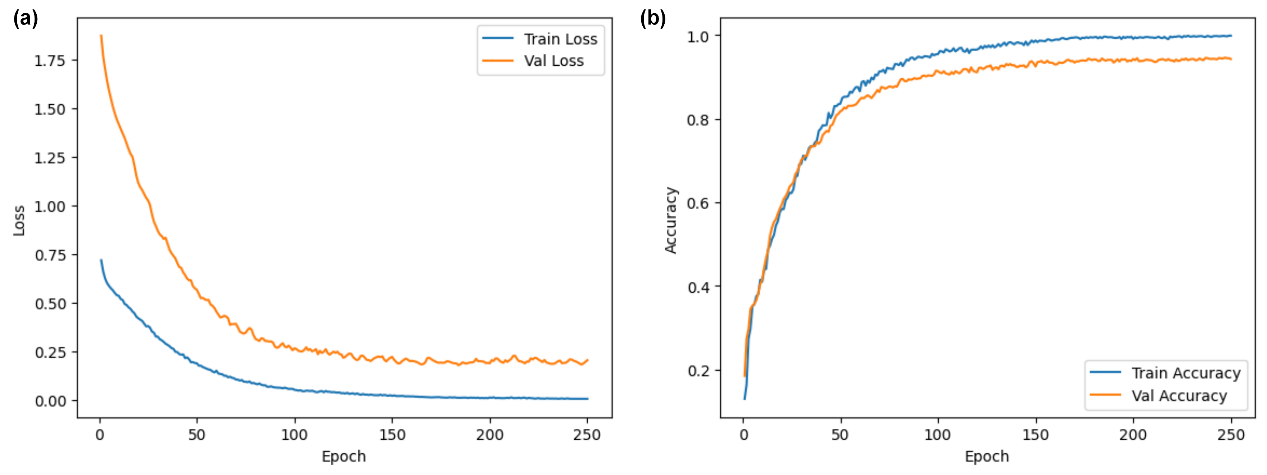


**Figure S21.** (a) Training loss plots and (b) training accuracy of Transformer model for seven motion object recognition.、

**Table S1.** The [O]/[F] ratio of Ti_3_C_2_T_x_ MXene and alk-Ti_3_C_2_T_x_ MXene measured by SEM-EDS and XPS, respectively.

| Measurement and characterization methods | Ti_3_C_2_T_x_ | Alk-Ti_3_C_2_T_x_ |
| --- | --- | --- |
| SEM-EDS | 3.80 | 10.53 |
| XPS | 2.28 | 10.92 |

**Table S2.** The comparison of sensitivity of wearable triboelectric pressure sensors in this article and previous articles.

| Sensitive material | Working mode | Sensitivity | Reference |
| --- | --- | --- | --- |
| ZnS/Cu/ polydimethylsiloxane | Vertical contact separation | 2 V/N | [1] |
| Microcrystalline cellulose/chitosan | Vertical contact separation | 2.2 V/N | [2] |
| Wrinkled polydimethylsiloxane/MXene | Single electrode | 1.8×10^-4^ V/kPa | [3] |
| Thermoplastic polyurethanes/polytetrafluoroethylene | Single electrode | 0.14 V/N | [4] |
| Silicon rubber | Vertical contact separation | 0.37 V/kPa | [5] |
| BaTiO_3_/ polydimethylsiloxane | Single electrode | 0.75 V/kPa | [6] |
| Micro-frustum-array polydimethylsiloxane | Vertical contact separation | 0.00567 V/kPa | [7] |
| Graphite/polyurethane | Single electrode | 0.13248 V/kPa | [8] |
| Cs_3_Sb_2_Cl_3_I_6_ | Vertical contact separation | 0.141 V/kPa | [9] |
| FG-P(AM-co-SMA) | Single electrode | 0.205 V/kPa | [10] |
| Au/peroxyacetyl nitrate  Au/nylon-66 | Vertical contact separation | 0.217V/kPa | [11] |
| Polycaprolactone CSYFs | Vertical contact separation | 0.388 V/kPa | [12] |
| MnTe_2_ | Vertical contact separation mode | 0.408 V/kPa | [13] |
| AFGO-embedded polyurethanes | Vertical contact separation | 0.0095 V/kPa | [14] |
| poly(diallyldimethylammonium chloride) (poly-DADMAC)/nylon-11 | Vertical contact separation | 1.01 V/kPa | [15] |
| Polydimethylsiloxane | Vertical contact separation | 1.04 V/kPa | [16] |
| a-MP@PP-8 | Single electrode | 1.48 V/kPa  4.7 V/N | This work |

**Reference**

[1] X. Zhang, Z. Li, W. Du, Y. Zhao, W. Wang, L. Pang, L. Chen, A. Yu, J. Zhai, *Nano Energy* **2022**, *96*, https://doi.org/10.1016/j.nanoen.2022.107115.

[2] J. Su, Y. Gao, Y. Yang, P. Fan, Z. Zhou, Z. Wang, X. Zhang, L. Fang, *ACS Appl Mater & Interfaces* **2024**, *16* (10), 12417, https://doi.org/10.1021/acsami.3c17241.

[3] Y. W. Cai, X. N. Zhang, G. G. Wang, G. Z. Li, D. Q. Zhao, N. Sun, F. Li, H. Y. Zhang, J. C. Han, Y. Yang, *Nano Energy* **2021**, *81*, https://doi.org/ARTN 105663

10.1016/j.nanoen.2020.105663.

[4] Y. F. Pang, X. Y. Zhu, S. N. Liu, C. K. Lee, *ACS Nano* **2023**, *17* (21), 21878, https://doi.org/10.1021/acsnano.3c08102.

[5] S. Lv, X. Zhang, T. Huang, H. Yu, M. Zhu, *Nano Energy* **2021**, *89*, https://doi.org/10.1016/j.nanoen.2021.106476.

[6] Y. Zhong, J. Wang, L. Wu, K. Liu, S. Dai, J. Hua, G. Cheng, J. Ding, *ACS Appl Mater & Interfaces* **2023**, *16* (1), 1727, https://doi.org/10.1021/acsami.3c14015.

[7] J. B. Yu, X. J. Hou, J. He, M. Cui, C. Wang, W. P. Geng, J. L. Mu, B. Han, X. J. Chou, *Nano Energy* **2020**, *69*, 11, https://doi.org/10.1016/j.nanoen.2019.104437.

[8] X. Gao, F. Xing, X. Hang, F. Guo, J. Wen, W. Sun, H. Song, Z. Lin Wang, B. Chen, *Chem. Eng. J.* **2024**, *493*, https://doi.org/10.1016/j.cej.2024.152645.

[9] L. Ding, Z. Wei, N. Sun, Y. Cai, Y. Zhou, K. Fang, G. Wang, *Chem. Eng. J.* **2024**, *487*, https://doi.org/10.1016/j.cej.2024.150395.

[10] R. Yan, Q. Sun, X. Shi, Z. Sun, S. Tan, B. Tang, W. Chen, F. Liang, H.-D. Yu, W. Huang, *Nano Energy* **2023**, *118*, https://doi.org/10.1016/j.nanoen.2023.108932.

[11] X. Peng, K. Dong, C. Ning, R. Cheng, J. Yi, Y. Zhang, F. Sheng, Z. Wu, Z. L. Wang, *Adv. Funct. Mater.* **2021**, *31* (34), https://doi.org/10.1002/adfm.202103559.

[12] M. Zhou, F. Xu, L. Ma, Q. Luo, W. Ma, R. Wang, C. Lan, X. Pu, X. Qin, *Nano Energy* **2022**, *104*, https://doi.org/10.1016/j.nanoen.2022.107885.

[13] C. C. Gowda, R. Tromer, D. Chandravanshi, P. Pandey, K. Chattopadhyay, D. S. Galvao, C. S. Tiwary, *Nano Energy* **2023**, *117*, 10, https://doi.org/10.1016/j.nanoen.2023.108833.

[14] G. Prasad, J. U. Yoon, I. Woo, J. W. Bae, *Chem. Eng. J.* **2023**, *470*, https://doi.org/10.1016/j.cej.2023.144280.

[15] S. M. S. Rana, M. T. Rahman, S. Sharma, M. Salauddin, S. H. Yoon, C. Park, P. Maharjan, T. Bhatta, J. Y. Park, *Nano Energy* **2021**, *88*, https://doi.org/10.1016/j.nanoen.2021.106300.

[16] S. Lee, J.-W. Park, *Nano Energy* **2022**, *101*, https://doi.org/10.1016/j.nanoen.2022.107546.
